# Identification of a founder *BRCA2* mutation in Sardinia

**DOI:** 10.1054/bjoc.1999.0963

**Published:** 2000-01-18

**Authors:** M Pisano, A Cossu, I Persico, G Palmieri, A Angius, G Casu, G Palomba, M G Sarobba, P C Ossu Rocca, M F Dedola, N Olmeo, A Pasca, M Budroni, V Marras, A Pisano, A Farris, G Massarelli, M Pirastu, Francesco Tanda

**Affiliations:** Istituto di Genetica Molecolare, CNR. Casella Postale, Santa Maria La Palma, Sassari, 07040, Italy; Istituto di Anatomia Patologica, Oncologia Medica and Sezione di Radioterapia, Università di Sassari, Viale San Pietro, Sassari, 07100, Italy; Oncologia Medica, II Laboratorio and Osservatorio Epidemiologico, Azienda U.S.L. n°1, Reg. S. Camillo, Sassari, 07100, Italy; Divisione Oncologica Medica 2, Ospedale Oncologico A. Businco. ASL 8, Via Jenner, Cagliari, 09100, Italy

**Keywords:** breast cancer, *BRCA2*, identity-by-descent, mutation, founder effect

## Abstract

Sardinian population can be instrumental in defining the molecular basis of cancer, using the identity-by-descent method. We selected seven Sardinian breast cancer families originating from the northern-central part of the island with multiple affected members in different generations. We genotyped 106 members of the seven families and 20 control nuclear families with markers flanking *BRCA2* locus at 13q12–q13. The detection of a common haplotype shared by four out of seven families (60%) suggests the presence of a founder *BRCA2* mutation. Direct sequencing of *BRCA2* coding exons of patients carrying the shared haplotype, allowed the identification of a ‘frame-shift’ mutation at codon 2867 (8765delAG), causing a premature termination-codon. This mutation was found in breast cancer patients as well as one prostate and one bladder cancer patient with shared haplotype. We then investigated the frequency of 8765delAG in the Sardinian breast cancer population by analysing 270 paraffin-embedded normal tissue samples from breast cancer patients. Five patients (1.7%) were found to be positive for the 8765delAG mutation. Discovery of a founder mutation in Sardinia through the identity-by-descent method demonstrates that this approach can be applied successfully to find mutations either for breast cancer or for other types of tumours. © 2000 Cancer Research Campaign


British Journal of Cancer (2000) **82**(3), 553–559
© 2000 Cancer Research Campaign
DOI: 10.1054/ bjoc.1999.0963, available online at http://www.idealibrary.com on IDEAL®

### Identification of a founder *BRCA2* mutation in Sardinia

**M Pisano[1,*], A Cossu[2,*], I Persico[1], G Palmieri[1], A Angius[1], G Casu[1], G Palomba[1], MG Sarobba[3], PC Ossu Rocca[2], MF Dedola[4], N Olmeo[5], A Pasca[6], M Budroni[7], V Marras[2], A Pisano[8], A Farris[3], G Massarelli[2], M Pirastu[1] and Francesco Tanda[2]**

[1]Istituto di Genetica Molecolare, CNR. Casella Postale, Santa Maria La Palma, 07040 Sassari, Italy; [2]Istituto di Anatomia Patologica, [3]Oncologia Medica and [4]Sezione di Radioterapia, Università di Sassari, Viale San Pietro, 07100 Sassari, Italy; [5]Oncologia Medica, [6]II Laboratorio and [7]Osservatorio Epidemiologico, Azienda U.S.L. n°1, Reg. S. Camillo, 07100 Sassari, Italy; [8]Divisione Oncologica Medica 2, Ospedale Oncologico A. Businco. ASL 8. Via Jenner, 09100 Cagliari, Italy

**Summary** Sardinian population can be instrumental in defining the molecular basis of cancer, using the identity-by-descent method. We selected seven Sardinian breast cancer families originating from the northern-central part of the island with multiple affected members in different generations. We genotyped 106 members of the seven families and 20 control nuclear families with markers flanking *BRCA2* locus at 13q12–q13. The detection of a common haplotype shared by four out of seven families (60%) suggests the presence of a founder *BRCA2* mutation. Direct sequencing of *BRCA2* coding exons of patients carrying the shared haplotype, allowed the identification of a 'frame-shift' mutation at codon 2867 (8765delAG), causing a premature termination-codon. This mutation was found in breast cancer patients as well as one prostate and one bladder cancer patient with shared haplotype. We then investigated the frequency of 8765delAG in the Sardinian breast cancer population by analysing 270 paraffin-embedded normal tissue samples from breast cancer patients. Five patients (1.7%) were found to be positive for the 8765delAG mutation. Discovery of a founder mutation in Sardinia through the identity-by-descent method demonstrates that this approach can be applied successfully to find mutations either for breast cancer or for other types of tumours. © 2000 Cancer Research Campaign

**Keywords**: breast cancer; *BRCA2*; identity-by-descent; mutation; founder effect

Breast cancer is the most common malignancy in women, with an incidence that varies between 40 and 90 per 100 000 (standardized rate) worldwide. Breast cancer is the most frequent female tumour in Italy, representing about 25% of all female tumours as reported in Italian registries (Zanetti et al, 1997).

A positive family history is known to be a high risk factor for developing the disease: 5–10% of all breast cancers arise in individuals carrying a germline mutation and are usually considered hereditary forms (Claus et al, 1991). Two major breast cancer-susceptibility genes, *BRCA1* and *BRCA2*, have been cloned (Miki et al, 1994; Wooster et al, 1995) and both are thought to account for 30–60% of hereditary breast cancer (Serova et al, 1997; Szabo et al, 1997; Vehmanen et al, 1997*a*, 1997*b*). However, large-scale mutation analyses conducted in several populations suggest the existence of additional breast cancer-susceptibility gene(s). *BRCA1* mutations are responsible for the majority of familial breast cancer associated with ovarian carcinoma, for about 50% of cases with breast cancer alone and for very few male breast cancer cases (Easton et al, 1993; Stratton et al, 1994; Narod et al, 1995). It has been estimated that women carrying a germline mutation in *BRCA1* have a risk ranging from 80 to 90% for developing breast cancer and from 44 to 63% for developing ovarian cancer (Easton et al, 1993, 1995; Ford et al, 1994; Miki et al, 1994; Wooster et al, 1994). *BRCA2* mutations account for a similar proportion of inherited breast cancer and are frequently associated with male breast cancer (Wooster et al, 1995). Breast cancer risk in females carrying *BRCA2* mutations is calculated to be similar to that conferred by *BRCA1* mutations (Easton et al, 1993, 1997; Ford et al, 1994; Miki et al, 1994; Wooster et al, 1994). *BRCA1* and particularly *BRCA2* families are often affected by other tumours such as prostate, liver, pancreas, lung, stomach and colorectum (Wooster et al, 1995; Gudmundsson et al, 1996; Phelan et al, 1996; Thorlacius et al, 1996; Vehmanen et al, 1997b; Tonin et al, 1998). Except for higher incidences of ovarian cancer in families with mutations in a 3.3-kb region of exon 11 of *BRCA2* (the so-called ovarian cancer cluster region [OCCR]; Gayther et al, 1997), no other significant association between genotype and phenotype was described. *BRCA1* and *BRCA2* mutations are for the most part frame-shifts due to small deletions leading to premature translation termination (Wooster et al, 1995; Phelan et al, 1996; Tavtigian et al, 1996; Gayther et al, 1997).

Some of these mutations are prevalent in genetically homogeneous populations as a consequence of a founder effect. A single *BRCA2* mutation accounts for the majority of hereditary breast cancer in Iceland (Gudmundsson et al, 1996; Thorlacius et al, 1996) and for 40% of male breast cancer cases (Johannesdottir et al, 1996), whereas three different founder mutations (185delAG and 5382insC in *BRCA1*, and 6174delT in *BRCA2*) have a high frequency in Ashkenazi Jews (Roa et al, 1996). Although at different rates, *BRCA1* and *BRCA2* founder mutations have been detected in other genetically homogeneous populations, such as the Finns (Vehmanen et al, 1997*a*) and the French-Canadians

*Received 6 May 1999*
*Revised 12 August 1999*
*Accepted 12 August 1999*

*Correspondence to:* M Pirastu

*These authors contributed equally.

(Tonin et al, 1998). In Sardinia, epidemiological data from the Regional Tumor Registry (accounting for the northern part of the island) indicate that breast carcinoma is the principal death-causing malignancy, with an incidence of 93 per 100 000 inhabitants (standardized rate) (Budroni et al, 1998). Sardinian population is genetically separated from that of the rest of Italy as well as from other European populations, due to strong genetic drift. All the monogenic disorders analysed, such as thalassemia (Pirastu et al, 1987), seem to be associated with a single founder mutation throughout the island. Therefore, it seems possible that such a founder effect could also be identified for a complex disease like cancer. This can be done by tracing back the mutation by linkage disequilibrium with genetic markers which give rise to a shared haplotype among the patients. On this basis, we decided to analyse the *BRCA2* gene using the identity-by-descent method, which allowed the identification of a mutation with founder effect in Sardinian breast cancer families.

### MATERIALS AND METHODS

### Breast cancer patients

Collaborating physicians at both the Department of Medical Oncology and the Institute of Histo-Pathology at Sassari University collected seven Sardinian families. Family ascertainment was carried out using the following criteria: (a) families with at least two affected members in different generations (either a first-degree relative or relative affected before age 50), or (b) families with at least three affected members. Clinical information was obtained from medical records.

Families were all apparently unrelated and originated from different small villages located in the northern-central part of the island; none of them presented cases of ovarian cancer. Three families had other forms of cancer (Figure 1). No breast cancer was detected in male members of the pedigrees. Blood samples were collected from 17 affected (15 breast cancer, one prostate cancer and one bladder cancer) and 89 unaffected members.

Twenty unrelated nuclear families, originating from the same geographical area with no history of breast cancer, were used as controls for the haplotype study.

Paraffin-embedded normal tissues were obtained from 270 breast cancer patients consecutively collected during 1997. No additional selection criteria were used to enrol patients in the screening; all cases were included regardless of age of onset. Sardinian origin was ascertained in all cases through genealogical studies. Informed consent was obtained from each family member before drawing blood.

### DNA analysis

DNA was isolated from blood samples using standard methods (Sambrook et al, 1989). DNA extraction from paraffin-embedded tissue was performed by a modification of the Jackson et al (1989) procedure. Briefly, single 7- to 8-mm tissue sections, cut from paraffin blocks, were stirred for 30 min with 1 ml of xylene in 1.5-ml tubes and centrifuged. The pellet was washed with ethanol, air-dried and resuspended in lysis solution (0.5% sodium dodecyl sulphate (SDS), 0.5 mg ml$^{-1}$ proteinase K in 1× TE buffer). After incubation at 37°C overnight and inactivation of proteinase K for 15 min, DNA was extracted with 1 vol of phenol, phenol–chloroform and chloroform. The supernatant was precipitated at –20°C overnight. The DNA was washed with 70% ethanol, air dried and resuspended in 10 mM Tris–HCl pH 7.5, 0.1 mM EDTA.

Polymorphic microsatellite markers used for haplotype analysis are linked to *BRCA2* gene at 13q12–q13 as reported in published genetic and physical maps: cen-D13S1246-D13S289-D13S260-D13S1698-*BRCA2*-D13S1701-D13S171-D13S267-D13S263-tel (Couch et al, 1996; Vehmanen et al, 1997*a*; Neuhausen et al, 1998; Marshmed map at http://www.marshmed.org). Polymerase chain reactions (PCR) were carried out as suggested in the Human Genome Database. PCR products were end-labelled with γ-$^{32}$P-dATP and electrophoresed on 6% acrylamide/7M urea sequencing gels. Alleles, visualized by X-ray autoradiography, were numbered according to size for each microsatellite repeat marker.

### *BRCA2* sequence analysis

Nucleotide sequencing of the entire *BRCA2* coding regions was initially performed in two patients with an identical haplotype belonging to two different families. DNA was amplified with primers specific for *BRCA2* exons (sequences and conditions are reported in the Human Genome Database). PCR products were gel purified using Qiaquick spin columns (Qiagen) and sequenced by Thermo Sequenase $^{33}$P-labelled terminator cycle sequencing kit (Amersham Pharmacia Biotech).

### Mutation screening

A new set of primers (Fd, 5′-GTGTAACACATTATTACAGTG-3′ and Rv, 5′-AATTCCTCCTGAATTTTAGTG-3′) was generated in order to bracket the region containing the mutation. Amplification conditions were: 94°C for 5 min, 30 cycles of 94°C for 30 s, 53°C for 30 s, 72°C for 1 min and 72°C for 10 min. Samples were electrophoresed on a 6% denaturing polyacrylamide gel for 2 h and the 2-bp deletion mutation was visualized as the faster migrating fragment after silver staining.

### RESULTS

Among the seven unrelated Sardinian families, 106 members, including 15 breast cancer cases, were genotyped with markers flanking the *BRCA2* locus at 13q12–q13. Pedigrees of all selected families are shown in Figure 1. The haplotypes generated with markers from D13S1246 to D13S1701 showed a pattern that was constant within each family for all affected members, as indicated in Figure 1. Four families shared the same haplotype in a 6.5 cM region from D13S1246 to D13S267. The haplotype shared by families 2 and 4 extended over a 15 cM interval reaching D13S263, the most telomeric marker (Figure 1). Interestingly, this haplotype was not detected in 80 control chromosomes except for alleles 11 and 2 of D13S171 and D13S267, respectively, found to be in linkage disequilibrium in the general Sardinian population (data not shown and Figure 1).

Patients III:18 of family 2 and patient III:6 of family 4 were affected by bladder and prostate cancer respectively. They shared the common extended haplotype of breast cancer patients. DNA samples from other family members with different tumours, including prostate, bladder, colorectal, gastric and brain (Figure 1) were unfortunately not available for analysis.

Two unrelated affected members carrying the common extended haplotype (patient III:7 of family 1 and II:2 of family 2; Figure 1) were chosen for direct sequencing of the *BRCA2* coding

 *© 2000 Cancer Research Campaign*

**Table 1** Mutational screening of unselected breast cancer patients.

| Age | No. of patients | Patients positive to 8765delAG (age at diagnosis) | Additional cancer cases in positive families |
|---|---|---|---|
| ≤ 40 | 42 | 1 (39) | 5 breast (1 male), 1 lung |
| 41–60 | 120 | 1 (45) | 5 breast, 1 lung, 1 liver |
| | | 1 (57) | 1 breast, 1 lung, 1 colon |
| > 60 | 108 | 1 (69) | 2 breast, 1 colon, 1 lung, 1 larynx |
| | | 1 (80) | 2 breast, 1 rectum |

The 270 consecutively collected patients are grouped according to age at diagnosis. For the five positive cases we indicate the age of onset and the number and types of cancer present in their families.

region. An AG deletion was found in exon 20 at codon 2846 (8765delAG), in both patients (Figure 2). This mutation is predicted to produce a truncated protein at codon 2867. The 8765delAG mutation was found in the remaining patients of family 2 (patients III:2 and III:19) and family 1 (patient III:11) (Figure 1). Interestingly, in patient III:19 of family 1 (Figure 1), who carries a different haplotype (Figure 1) this mutation was not detected. After screening the other five families, patient III:5 of family 6 and patients III:7, III:12 and III:6 (prostate cancer) of family 4 as well as patient III:18 (bladder cancer) of family 2 were found positive for the presence of 8765delAG mutation as expected from the shared haplotype (Figure 1). Patients from families 3, 5 and 7 were negative for the 8765delAG mutation, confirming the haplotype results. Altogether this mutation was detected in four out of seven families (60%).

To investigate the frequency of the 8765delAG mutation, we analysed 270 paraffin-embedded normal tissues from breast cancer patients coming from the northern-central part of Sardinia. All cases were collected regardless of family history and age of onset. We found that five out of 270 patients (1.7%) carried this mutation (Table 1).

### DISCUSSION

Sardinia has a relatively small, isolated, and genetically homogeneous population with a high rate of inbreeding making it ideal for genetic studies on either monogenic or multifactorial disorders. Several founder effects have already been demonstrated for monogenic diseases in this population. Therefore it seemed possible that founder mutations could also be detected in cancer patients. Analysis of family pedigrees over several generations and use of polymorphic markers may identify a common identical-by-descent haplotype, in affected individuals. This strategy could help restricting the number of cases for mutation screening, avoiding extensive analysis principally when the candidate gene is as large as *BRCA2*.

In the seven breast cancer families with multiple affected members in different generations, selected for our study, clinical phenotype, absence of ovarian cancer and late age-of-onset suggested *BRCA2* as a candidate gene. Genotyping with markers flanking the *BRCA2* gene at 13q12–q13 locus identified a large haplotype in four out of seven families, not found in control chromosomes from the same geographical area. A few patients from each family were additionally genotyped with markers closely linked to the *BRCA1* gene at 17q21. We found no differences in haplotype frequency between patients and normal controls (data not shown). Presence of a founder mutation in the *BRCA2* gene was confirmed by identification of a 2-bp (AG) deletion in exon 20. This mutation is located outside of the OCCR region of the *BRCA2* gene, in agreement with ovarian cancer absence in our families. This AG deletion at nucleotide 8765 was already described as a founder mutation in Yemenite-Jews families (Lerer et al, 1998), as well as French-Canadian families (Phelan et al, 1996; Tonin et al, 1998). In order to understand if this mutation has a common ancestral origin we carried out a haplotype analysis of Sardinian and French-Canadian families (DNA samples of two French-Canadian 8765delAG carriers were kindly provided by P Tonin). This study showed that in the two populations the 8765delAG is associated to different haplotypes (data not shown). These results support the hypothesis that the 8765delAG mutation occurred at least twice in different populations because of its position in an AG-rich sequence which may be a mutational hot-spot.

The 8765delAG mutation was present in all affected individuals who shared the identical-by-descent haplotype. Unfortunately, some family members were not evaluated for such mutation due to individual refusal to undergo this analysis. Patient III:19 of family 1 and breast cancer patients from other families showing a different haplotype (Figure 1) were found to be negative for the 8765delAG mutation. Family 1 strikingly shows that two sisters (patients III:11 and III:19) do not have the same genotype: patient III:11, who carries the 8765delAG mutation, seems to have received it from the father's side (because the same mutation is present in family member III:7). For patient III:19, who does not carry this mutation, we can hypothesize that she either received another mutation from the mother's side (in which breast cancer is also present) or she is a phenocopy due to the high heterogenity of the genetic and non-genetic factors causing the disease. Actually, the young age of the third-generation relatives and missing genotype data on some family members (i.e. the affected maternal aunt) are hindering clarification of this point.

As reported in Figure 1, two patients with other tumours (bladder carcinoma for patient III:18 of family 2 and prostate carcinoma for patient III:6 of family 4) shared the same haplotype with the breast cancer patients. In these two cases, we also found the 8765delAG mutation. The association of other tumours with breast cancer is reported by several authors (Phelan et al, 1996; Thorlacius et al, 1996; Serova et al, 1997; Tonin et al, 1998) and is confirmed in our families. It would be interesting to carry out a screening for this mutation in families with familial bladder and prostate cancers.

The frequency of 8765delAG in Sardinia was subsequently verified by screening 270 breast cancer patients consecutively

© 2000 Cancer Research Campaign 

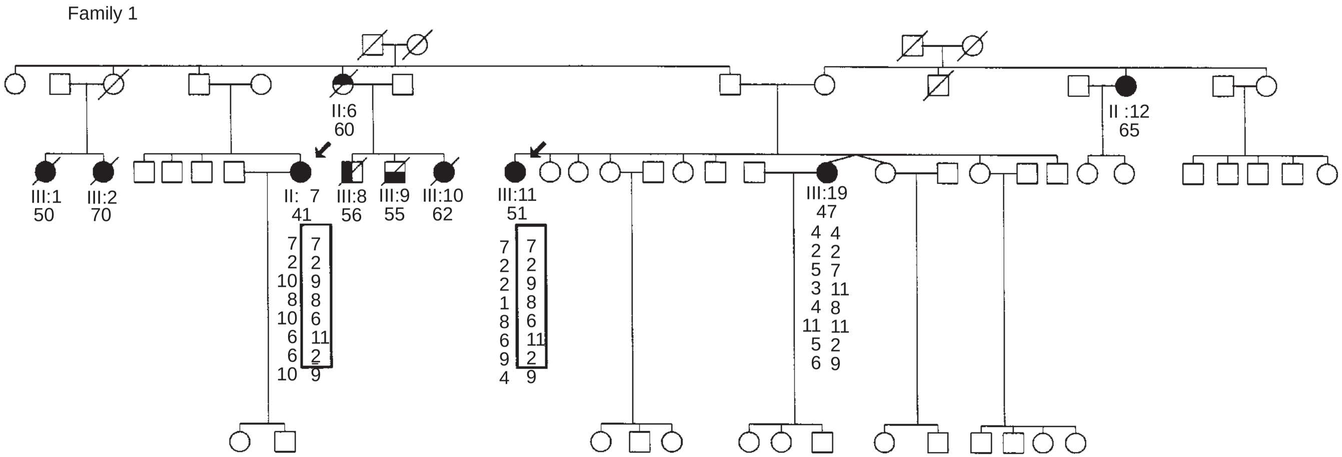

| Marker | Position (cM) | Alleles | Het. (%) |
|---|---|---|---|
| D13S1246 | 3.4 | 1..10 | 82 |
| D13S289 | 2.3 | 1..12 | 74 |
| D13S260 | 0.8 | 1..10 | 78 |
| D13S1698 | 0.2 | 1..15 | 63 |
| **BRCA2** | **0** | | |
| D13S1701 | −0.5 | 1..18 | 88 |
| D13S171 | −0.6 | 1..11 | 72 |
| D13S267 | −3.1 | 1..15 | 69 |
| D13S263 | −12 | 1..12 | 84 |

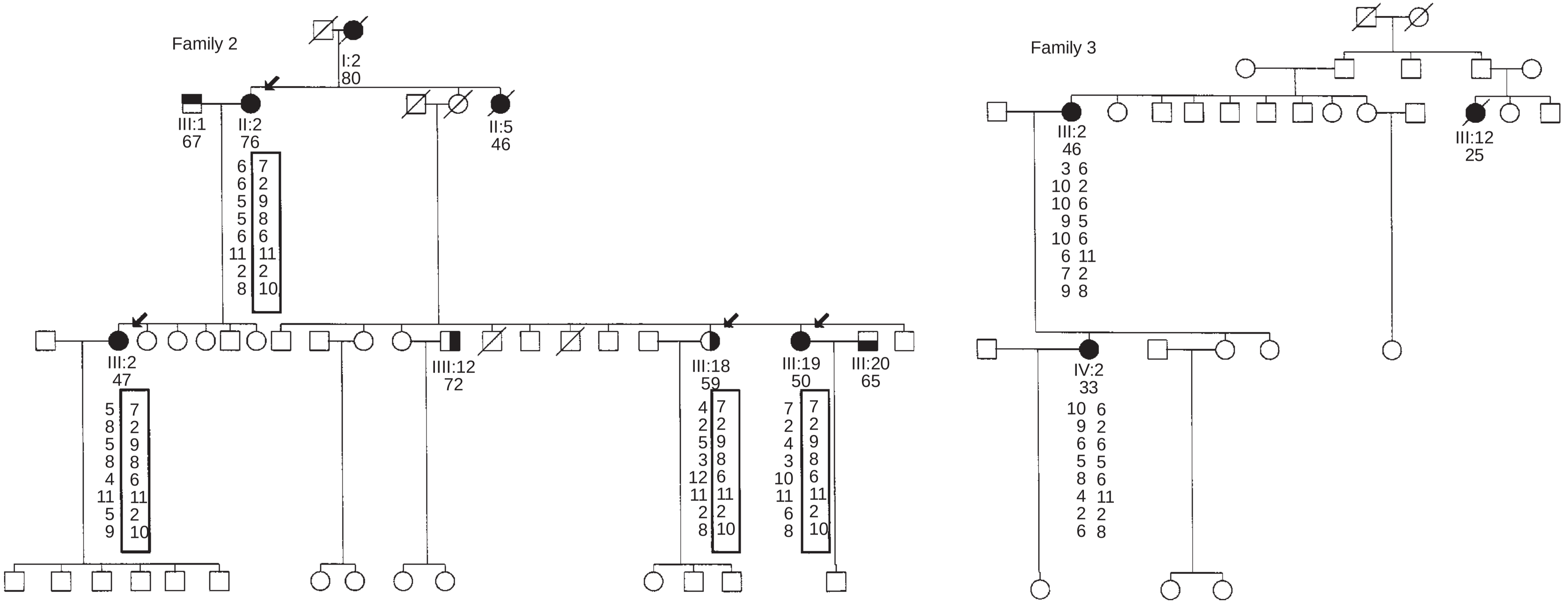

**Figure 1** Pedigrees and haplotype results. All family members for each pedigree are included. Symbol definitions are as follows: □, ○ unaffected; ■, ● breast cancer; ◧ prostate cancer; ◨, ◑ bladder cancer; ⬓, ⊖ colorectal cancer; ⬒, ◓ liver cancer; ⧉, ⊕ brain cancer. Each affected individual is indicated with the generation identifier, age at diagnosis and specific haplotype. Shared haplotypes are boxed. Arrows indicate the cases carrying the 8765delAG mutation.

*© 2000 Cancer Research Campaign*

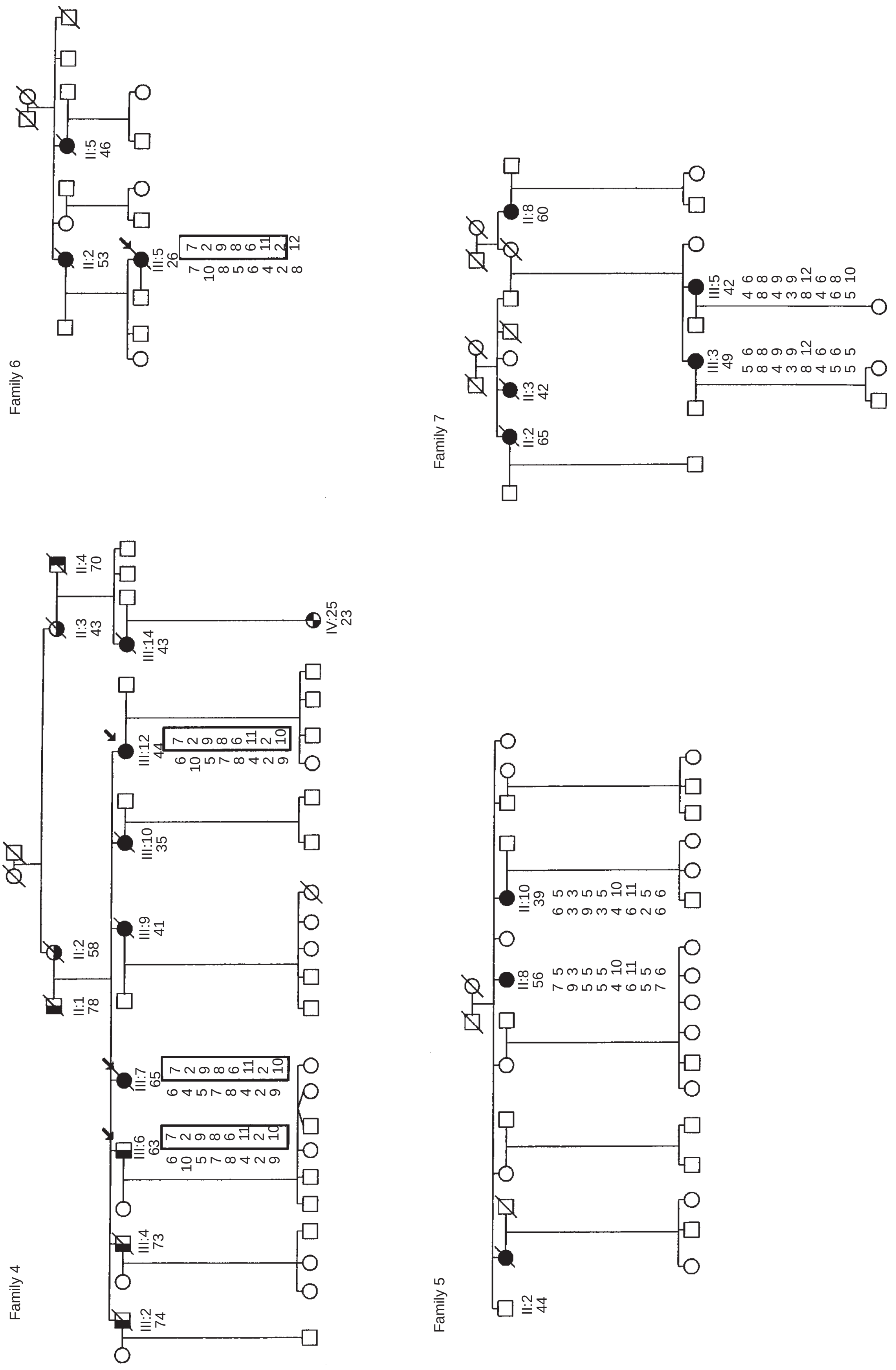

**Figure 1** *continued*

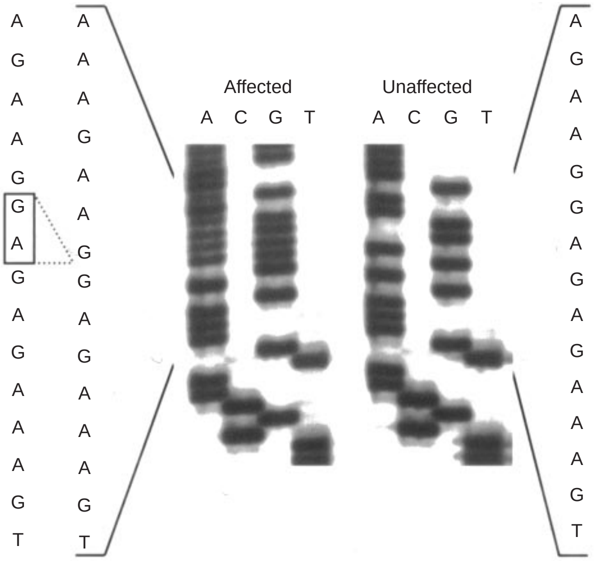

**Figure 2** Deletion sequence. Sequencing of affected and unaffected individuals are compared. Boxed letters represent deleted nucleotides in affected individuals collected over a 1-year period, regardless of family history or age of onset, from the central-northern part of the island. We found five patients positive to the 8765delAG mutation with a frequency of 1.7% in this group of unselected patients (see Table 1). Preliminary results of a similar screening conducted in breast cancer patients from the southern part of the island indicate that 8765delAG mutation is present at a lower frequency (1/208; 0.5%).

The discrepancy in the frequency of the 8765delAG mutation between familial (60%) and unselected cases (1.7%) probably is due to the selection criteria used. Families were selected on the basis of the presence of several affected cases in different generations segregating a dominant trait with high penetrance. On the contrary, the mutation screening was carried out in unselected cases of which only 5–10% are expected to be familial (Claus et al, 1991). In addition, as already shown in several similar studies carried out in different populations, only a small proportion of familial cases are due to *BRCA2* defects. As a confirmation of this, we found that the five patients (unrelated to our cohort of families) carrying the 8765delAG mutation, came from families with multiple cases of breast cancer as well as other tumours in first- and second-degree relatives (data not shown). These data again support the value of screening for this mutation in Sardinia. Searching for the 8765delAG mutation in these kind of families would be important for the detection of asymptomatic carriers. Additional studies on the three 8765delAG-negative families could help identifying new *BRCA2* mutations in our population.

Although described as a founder mutation in other populations (Lerer et al, 1998; Tonin et al, 1998), the 8765delAG may be considered the first *BRCA2* founder in Italian breast cancer families. Most Italian breast cancer cases are *BRCA1*-linked with a high incidence of familial ovarian cancer and without any evidence of a founder effect (De Benedetti et al, 1996; Montagna et al, 1996). An intriguing hypothesis is that founder effects might be detected in other Italian regions by selection of families coming from small geographical areas showing genetic micro-homogeneity. Indeed, the approach used in our study clearly demonstrates that in multifactorial diseases like cancer, founder effects are likely to be detected when the population studied is relatively small and genetically homogeneous. Identification of prevalent mutations in such populations could represent an essential pre requisite for a prevention programme based on DNA analysis.

### ACKNOWLEDGEMENTS

We gratefully acknowledge patients and their families for their important contribution to this study. This study was supported by a grant from Assessorato dell'Igiene e Sanità e dell'Assistenza Sociale, Regione Autonoma della Sardegna.

### REFERENCES

Budroni M, Cesaraccio R, Desole MG, Pirino DR, Sechi O, Massarelli G, Tanda F, Manca A, Cossu Rocca P and Cocco L (1998) Incidenza dei tumori nella provincia di Sassari Anni 1992–1994. In: *Incidenza e mortalità per tumori nella provincia di Sassari Anni 1992–1994*, Budroni M and Tanda F (eds). Tipografia Moderna: Sassari

Claus EB, Risch N and Thompson WD (1991) Genetic analysis of breast cancer in the Cancer and Steroid Hormone Study. *Am J Hum Genet* **48:** 232–242

Couch FJ, Rommens JM, Neuhausen SL, Belanger C, Dumont M, Abel K, Bell R, Berry S, Bogden R, Cannon-Albright L, Farid L, Frye C, Hattier T, Janecki T, Jiang P, Kehrer R, Leblanc JF, McArthur-Morrison J, McSweeney D, Miki Y, Peng Y, Samson C, Schroeder M, Snyder SC, Stringfellow M, Stroup C, Swedlund B, Swensen J, Teng D, Thakur S, Tran T, Tranchant M, Welver-Feldhaus J, Wong AKC, Shizuya H, Labrie F, Skolnick MH, Goldgar DE, Kamb A, Weber BL, Tavtigian SV and Simard J (1996) Generation of an integrated transcription map of the *BRCA2* region on chromosome 13q12–q13. *Genomics* **36**: 86–99

De Benedetti VMG, Radice P, Mondini P, Spatti G, Conti A, Illeni MT, Caligo MA, Cipollini G, Bevilaqua G, Pilotti S and Pierotti MA (1996) Screening for mutations in exon 11 of the *BRCA1* gene in 70 Italian breast and ovarian cancer patients by protein truncation test. *Oncogene* **13**: 1353–1357

Easton DF, Bishop DT, Ford D, Crockford GP and the Breast Cancer Linkage Consortium (1993) Genetic linkage analysis in familial breast and ovarian cancer: results from 214 families. *Am J Hum Genet* **52**: 678–701

Easton DF, Ford D, Bishop DT and the Breast Cancer Linkage Consortium (1995) Breast and ovarian cancer incidence in *BRCA1*-mutation carriers. *Am J Hum Genet* **56**: 265–271

Easton DF, Steele L, Fields P, Ormiston W, Averill D, Daly PA, McManus R, Neuhausen SL, Ford D, Wooster R, Cannon-Albright LA, Stratton MR and Goldgar DE (1997) Cancer risks in two large breast cancer families linked to *BRCA2* on chromosome 13q12–13. *Am J Hum Genet* **61**: 120–128

Ford D, Easton DF, Bishop DT, Narod SA, Goldgar DT and the Breast Cancer Linkage Consortium (1994) Risks of cancer in *BRCA1* mutation carriers. *Lancet* **343**: 692–695

Gayther SA, Mangion J, Russel P, Seal S, Barfoot R, Ponder BAJ, Stratton MR and Easton D (1997) Variation of risks of breast and ovarian cancer associated with different germline mutations of the *BRCA2* gene. *Nat Genet* **15**: 103–105

Gudmundsson J, Johannesdottir G, Arason A, Bergthorsson JT, Ingvarsson S, Egilsson V and Barkadottir RB (1996) Frequent occurrence of *BRCA2* linkage in Icelandic breast cancer families and segregation of a common *BRCA2* haplotype. *Am J Hum Genet* **58**: 749–756

Jackson DP, Quirke P, Lewis F, Boylston AW, Sloan JM, Robertson D and Taylor GR (1989) Detection of measles virus RNA in paraffin-embedded tissue. *Lancet* **17**: 1391

Johannesdottir G, Gudmundsson J, Bergthorsson JT, Arason A, Agnarsson BA, Eiriksdottir G, Johannsson OT, Borg A, Ingvarsson S, Easton DF, Egilsson V and Barkardottir RB (1996) High prevalence of the 999del5 mutation in icelandic breast and ovarian cancer patients. *Cancer Res* **56**: 3663–3665

Lerer I, Wang T, Peretz T, Sagi M, Kaduri L, Orr-Urtreger A, Stadler J, Gutman H and Abeliovich D (1998) The 8765delAG mutation in *BRCA2* is common among Jews of Yemenite extraction. *Am J Hum Genet* **63**: 274–279

Miki Y, Swensen J, Schattuck-Eidens D, Futreal PA, Harshman K, Tavtigian S, Liu QY, Cochran C, Bennet LM, Ding W, Bell R, Rosenthal J, Hussey C, Tran T, McClure M, Frye C, Hattier T, Phelps R, Haugen-Strano A, Katcher H, Yakumo K, Gholami Z, Shaffer D, Stone S, Bayer S, Wray C, Bogden R,

 *© 2000 Cancer Research Campaign*

Dayananth P, Ward J, Tonin P, Narod S, Bristow PK, Norris FH, Helvering L, Morrison P, Rosteck P, Lai M, Barret JC, Lewis C, Neuhausen S, Cannon-Albright L, Goldgar D, Wiseman R, Kamb A and Skolnick MH (1994) A strong candidate for the breast and ovarian cancer susceptibility gene *BRCA1*. *Science* **266**: 66–71

Montagna M, Santacatterina M, Corneo B, Menin C, Serova O, Lenoir GM, Chieco-Bianchi L and D'Andrea E (1996) Identification of seven new *BRCA1* germline mutations in Italian breast and breast/ovarian cancer families. *Cancer Res* **56**: 5466–5469

Narod SA, Ford D, Devilee P, Barkadottir RB, Lynch HT, Smith SA, Ponder BA, Weber BL, Garber JE, Birch JM, Cornelis RS, Kelsell DP, Spurr NK, Smyth E, Haites N, Sobol H, Bignon Y-J, Chang-Claude J, Hamann U, Lindblom A, Borg A, Piver MS, Gallion HH, Struewing JP, Whittemore A, Tonin P, Goldgar DE, Easton DF and the Breast Cancer Linkage Consortium (1995) An evaluation of genetic heterogeneity in 145 breast-ovarian cancer families. *Am J Hum Genet* **56**: 254–264

Neuhausen SL, Godwin AK, Gershoni-Baruch R, Schubert E, Garber J, Stoppa-Lyonnet D, Olah E, Csokay B, Serova O, Lalloo F, Osorio A, Stratton M, Offit K, Boyd J, Caligo MA, Scott RJ, Schofield A, Teugels E, Schwab M, Cannon-Albright L, Bishop T, Easton D, Benitez J, King MC, Ponder BAJ, Weber B, Devilee P, Borg A, Narod SA and Goldgar D (1998) Haplotype and phenotype analysis of nine recurrent *BRCA2* mutations in 111 families: results of an international study. *Am J Hum Genet* **62**: 1381–1388

Phelan CM, Lancaster JM, Tonin P, Gumbs C, Cochran C, Carter R, Ghadirian P, Perret C, Moslehi R, Dion F, Faucher MC, Dole K, Karimi S, Foulkes W, Lounis H, Warner E, Goss P, Anderson D, Larsson C, Narod SA and Futreal PA (1996) Mutation analysis of the *BRCA2* gene in 49 site-specific breast cancer families. *Nat Genet* **13**: 120–122

Pirastu M, Galanello R, Doherty MA, Tuveri T, Cao A and Kan YW (1987) The same β-globin gene mutation is present on nine different β-thalassemia chromosomes in a Sardinian population. *Proc Natl Acad Sci USA* **84**: 2882–2885

Roa BB, Boyd AA, Volcik K and Richards CS (1996) Ashkenazi Jewish population frequencies for common mutations in *BRCA1* and *BRCA2*. *Nat Genet* **14**: 185–187

Sambrook J, Fritsch EF and Maniatis T (1989) *Molecular Cloning: A Laboratory Manual*. Cold Spring Harbor Laboratory Press: Cold Spring Harbor

Serova OM, Mazoyer S, Puget N, Dubois V, Tonin P, Shugart YY, Goldgar D, Narod SA, Lynch HT and Lenoir GM (1997) Mutations in *BRCA1* and *BRCA2* in breast cancer families: are there more breast cancer-susceptibility genes? *Am J Hum Genet* **60**: 486–495

Stratton MR, Ford D, Neuhausen S, Seal S, Wooster R, Friedman LS, King M-C, Egilsson V, Devilee P, McManus R, Daly PA, Smyth E, Ponder BAJ, Peto J, Cannon-Albright L, Easton DF and Goldgar DE (1994) Familial male breast cancer is not linked to *BRCA1*. *Nat Genet* **7**: 103–107

Szabo CI and King MC (1997) Population genetics of *BRCA1* and *BRCA2*. *Am J Hum Genet* **60**: 1013–102

Tavtigian SV, Simard J, Rommens J, Couch F, Shattuck-Eidens D, Neuhausen S, Merajver S, Thorlacius S, Offit K, Stoppa-Lyonnet D, Belanger C, Bell R, Berry S, Bogden R, Chen Q, Davis T, Dumont M, Frye C, Hattier T, Jammulapati S, Janecki T, Jiang P, Keher R, Leblanc J-F, Mitchell JT, McArthur-Morrison J, Nguyen K, Peng Y, Samson C, Schroeder M, Snyder SC, Steele L, Stringfellow M, Stroup C, Swedlund B, Swensen J, Teng D, Thomas A, Tran T, Tranchant M, Weaver-Feldhaus J, Wong AKC, Shizuya H, Eyfiord J, Cannon-Albright L, Labrie F, Skolnick MH, Weber B, Kamb A and Goldgar DE (1996) The complete gene and mutations in chromosome 13q-linked kindreds. *Nat Genet* **12**: 333–337

Thorlacius S, Tryggvadottir L, Olafsdottir GH, Jonasson JG, Ogmundsdottir HM, Tulinius H and Eyfjord JE (1996) A single *BRCA2* mutation in male and female breast cancer families from Iceland with varied cancer phenotypes. *Nat Genet* **13**: 117–119

Tonin PN, Mes-Masson AM, Futreal PA, Morgan K, Mahon M, Foulkes WD, Cole DEC, Provencher D, Ghadirian P and Narod SA (1998) Founder *BRCA1* and *BRCA2* mutations in French Canadian breast and ovarian cancer families. *Am J Hum Genet* **63**: 1341–1351

Vehmanen P, Friedman LS, Eerola H, Sarantaus L, Pyrhönen S, Ponder B, Muhonen T and Nevanlinna H (1997*a*) A low proportion of *BRCA2* mutations in Finnish breast cancer families. *Am J Hum Genet* **60**: 1050–1058

Vehmanen P, Friedman LS, Eerola H, McClure M, Ward B, Sarantaus L, Kainu T, Syrjäkoski K, Pyrhönen S, Kallioniemi OP, Muhonen T, Luce M, Frank TS and Nevanlinna H (1997*b*) Low proportion of *BRCA1* and *BRCA2* mutations in Finnish breast cancer families: evidence for additional susceptibility genes. *Hum Mol Genet* **13**: 2309–2315

Wooster R, Bignell G, Lancaster J, Swift S, Seal S, Mangion J, Collins N, Gregory S, Gumbs C, Micklem G, Barfoot R, Hamoudi R, Patel S, Rice C, Biggs P, Hashim Y, Smith A, Connor F, Arason A, Gudmundsson J, Ficenec D, Kelsell D, Ford D, Tonin P, Bishop DT, Spurr NK, Ponder BAJ, Eeles R, Peto J, Devilee P, Cornelisse C, Lynch H, Narod S, Lenoir G, Egilsson B, Barkadottir RB, Easton DF, Bentley DR, Futreal PA, Ashworth A and Stratton MR (1995) Identification of the breast cancer susceptibility gene *BRCA2*. *Nature* **378**: 789–792

Zanetti R, Vercelli M, Crossignani P, Simonato L, Stanta G, Cocconi G, Federico N, Ferretti S, Amadori D, Pannelli F, Buiati E, Conti FMS, Gafà L, Magnani C, Ponz de Leon M and Picci P (1997) *Cancer in Italy. Incidence Data from Cancer Registries, vol. 2: 1988–1992*, Zanetti R, Crosignani P and Rosso S (eds). I1 Pensiero Scientifico Editore: Roma

*© 2000 Cancer Research Campaign*

## References

[PDF_00790] Claus E. B., Risch N., Thompson W. D. (1991). Genetic analysis of breast cancer in the cancer and steroid hormone study.. Am J Hum Genet.

[PDF_00797] Couch F. J., Rommens J. M., Neuhausen S. L., Bélanger C., Dumont M., Abel K., Bell R., Berry S., Bogden R., Cannon-Albright L. (1996). Generation of an integrated transcription map of the BRCA2 region on chromosome 13q12-q13.. Genomics.

[PDF_00801] De Benedetti V. M., Radice P., Mondini P., Spatti G., Conti A., Illeni M. T., Caligo M. A., Cipollini G., Bevilaqua G., Pilotti S. (1996). Screening for mutations in exon 11 of the BRCA1 gene in 70 Italian breast and ovarian cancer patients by protein truncation test.. Oncogene.

[PDF_00805] Easton D. F., Bishop D. T., Ford D., Crockford G. P. (1993). Genetic linkage analysis in familial breast and ovarian cancer: results from 214 families. The Breast Cancer Linkage Consortium.. Am J Hum Genet.

[PDF_00808] Easton D. F., Ford D., Bishop D. T. (1995). Breast and ovarian cancer incidence in BRCA1-mutation carriers. Breast Cancer Linkage Consortium.. Am J Hum Genet.

[PDF_00811] Easton D. F., Steele L., Fields P., Ormiston W., Averill D., Daly P. A., McManus R., Neuhausen S. L., Ford D., Wooster R. (1997). Cancer risks in two large breast cancer families linked to BRCA2 on chromosome 13q12-13.. Am J Hum Genet.

[PDF_00815] Ford D., Easton D. F., Bishop D. T., Narod S. A., Goldgar D. E. (1994). Risks of cancer in BRCA1-mutation carriers. Breast Cancer Linkage Consortium.. Lancet.

[PDF_00818] Gayther S. A., Mangion J., Russell P., Seal S., Barfoot R., Ponder B. A., Stratton M. R., Easton D. (1997). Variation of risks of breast and ovarian cancer associated with different germline mutations of the BRCA2 gene.. Nat Genet.

[PDF_00821] Gudmundsson J., Johannesdottir G., Arason A., Bergthorsson J. T., Ingvarsson S., Egilsson V., Barkardottir R. B. (1996). Frequent occurrence of BRCA2 linkage in Icelandic breast cancer families and segregation of a common BRCA2 haplotype.. Am J Hum Genet.

[PDF_00825] Jackson D. P., Quirke P., Lewis F., Boylston A. W., Sloan J. M., Robertson D., Taylor G. R. (1989). Detection of measles virus RNA in paraffin-embedded tissue.. Lancet.

[PDF_00828] Johannesdottir G., Gudmundsson J., Bergthorsson J. T., Arason A., Agnarsson B. A., Eiriksdottir G., Johannsson O. T., Borg A., Ingvarsson S., Easton D. F. (1996). High prevalence of the 999del5 mutation in icelandic breast and ovarian cancer patients.. Cancer Res.

[PDF_00832] Lerer I., Wang T., Peretz T., Sagi M., Kaduri L., Orr-Urtreger A., Stadler J., Gutman H., Abeliovich D. (1998). The 8765delAG mutation in BRCA2 is common among Jews of Yemenite extraction.. Am J Hum Genet.

[PDF_00893] Miki Y., Swensen J., Shattuck-Eidens D., Futreal P. A., Harshman K., Tavtigian S., Liu Q., Cochran C., Bennett L. M., Ding W. (1994). A strong candidate for the breast and ovarian cancer susceptibility gene BRCA1.. Science.

[PDF_00897] Montagna M., Santacatterina M., Corneo B., Menin C., Serova O., Lenoir G. M., Chieco-Bianchi L., D'Andrea E. (1996). Identification of seven new BRCA1 germline mutations in Italian breast and breast/ovarian cancer families.. Cancer Res.

[PDF_00910] Neuhausen S. L., Godwin A. K., Gershoni-Baruch R., Schubert E., Garber J., Stoppa-Lyonnet D., Olah E., Csokay B., Serova O., Lalloo F. (1998). Haplotype and phenotype analysis of nine recurrent BRCA2 mutations in 111 families: results of an international study.. Am J Hum Genet.

[PDF_00914] Phelan C. M., Lancaster J. M., Tonin P., Gumbs C., Cochran C., Carter R., Ghadirian P., Perret C., Moslehi R., Dion F. (1996). Mutation analysis of the BRCA2 gene in 49 site-specific breast cancer families.. Nat Genet.

[PDF_00919] Pirastu M., Galanello R., Doherty M. A., Tuveri T., Cao A., Kan Y. W. (1987). The same beta-globin gene mutation is present on nine different beta-thalassemia chromosomes in a Sardinian population.. Proc Natl Acad Sci U S A.

[PDF_00923] Roa B. B., Boyd A. A., Volcik K., Richards C. S. (1996). Ashkenazi Jewish population frequencies for common mutations in BRCA1 and BRCA2.. Nat Genet.

[PDF_00928] Serova O. M., Mazoyer S., Puget N., Dubois V., Tonin P., Shugart Y. Y., Goldgar D., Narod S. A., Lynch H. T., Lenoir G. M. (1997). Mutations in BRCA1 and BRCA2 in breast cancer families: are there more breast cancer-susceptibility genes?. Am J Hum Genet.

[PDF_00932] Stratton M. R., Ford D., Neuhasen S., Seal S., Wooster R., Friedman L. S., King M. C., Egilsson V., Devilee P., McManus R. (1994). Familial male breast cancer is not linked to the BRCA1 locus on chromosome 17q.. Nat Genet.

[PDF_00936] Szabo C. I., King M. C. (1997). Population genetics of BRCA1 and BRCA2.. Am J Hum Genet.

[PDF_00938] Tavtigian S. V., Simard J., Rommens J., Couch F., Shattuck-Eidens D., Neuhausen S., Merajver S., Thorlacius S., Offit K., Stoppa-Lyonnet D. (1996). The complete BRCA2 gene and mutations in chromosome 13q-linked kindreds.. Nat Genet.

[PDF_00948] Thorlacius S., Olafsdottir G., Tryggvadottir L., Neuhausen S., Jonasson J. G., Tavtigian S. V., Tulinius H., Ogmundsdottir H. M., Eyfjörd J. E. (1996). A single BRCA2 mutation in male and female breast cancer families from Iceland with varied cancer phenotypes.. Nat Genet.

[PDF_00952] Tonin P. N., Mes-Masson A. M., Futreal P. A., Morgan K., Mahon M., Foulkes W. D., Cole D. E., Provencher D., Ghadirian P., Narod S. A. (1998). Founder BRCA1 and BRCA2 mutations in French Canadian breast and ovarian cancer families.. Am J Hum Genet.

[PDF_00959] Vehmanen P., Friedman L. S., Eerola H., McClure M., Ward B., Sarantaus L., Kainu T., Syrjäkoski K., Pyrhönen S., Kallioniemi O. P. (1997). Low proportion of BRCA1 and BRCA2 mutations in Finnish breast cancer families: evidence for additional susceptibility genes.. Hum Mol Genet.

[PDF_00956] Vehmanen P., Friedman L. S., Eerola H., Sarantaus L., Pyrhönen S., Ponder B. A., Muhonen T., Nevanlinna H. (1997). A low proportion of BRCA2 mutations in Finnish breast cancer families.. Am J Hum Genet.

[PDF_00964] Wooster R., Bignell G., Lancaster J., Swift S., Seal S., Mangion J., Collins N., Gregory S., Gumbs C., Micklem G. (1995). Identification of the breast cancer susceptibility gene BRCA2.. Nature.

